# Anabolic and catabolic responses of human articular chondrocytes to varying oxygen percentages

**DOI:** 10.1186/ar2942

**Published:** 2010-03-02

**Authors:** Simon Ströbel, Marko Loparic, David Wendt, Andreas D Schenk, Christian Candrian, Raija LP Lindberg, Florina Moldovan, Andrea Barbero, Ivan Martin

**Affiliations:** 1 Departments of Surgery and of Biomedicine, University Hospital Basel, Hebelstrasse 20, Basel, 4031, Switzerland; 2 M.E. Müller Institute for Structural Biology, Biozentrum University of Basel, Klingelbergstrasse 50/70, Basel, 4056, Switzerland; 3 Department of Orthopaedic Surgery and Traumatology, Ospedale Regionale di Lugano, Via Tesserete 46, Lugano, 6900, Switzerland; 4 Departments of Biomedicine and Neurology, University Hospital Basel, Hebelstrasse 20, Basel, 4031, Switzerland; 5 Faculty of Dentistry and CHU Sainte-Justine, University of Montreal, 3175 Côte Sainte-Catherine, Montreal, H3T1C5, Canada

## Abstract

**Introduction:**

Oxygen is a critical parameter proposed to modulate the functions of chondrocytes ex-vivo as well as in damaged joints. This article investigates the effect of *low *(more physiological) oxygen percentage on the biosynthetic and catabolic activity of human articular chondrocytes (HAC) at different phases of *in vitro *culture.

**Methods:**

HAC expanded in monolayer were cultured in pellets for two weeks (Phase I) or up to an additional two weeks (Phase II). In each Phase, cells were exposed to 19% or 5% oxygen. Resulting tissues and culture media were assessed to determine amounts of produced/released proteoglycans and collagens, metalloproteinases (MMPs), collagen degradation products and collagen fibril organization using biochemical, (immuno)-histochemical, gene expression and scanning electron microscopy analyses. In specific experiments, the hypoxia-inducible factor-1α (HIF-1α) inhibitor cadmium chloride was supplemented in the culture medium to assess the involvement of this pathway.

**Results:**

Independent from the oxygen percentage during expansion, HAC cultured at 5% O_2 _(vs 19% O_2_) during Phase I accumulated higher amounts of glycosaminoglycans and type II collagen and expressed reduced levels of *MMP-1 *and *MMP-13 *mRNA and protein. Switching to 19% oxygen during Phase II resulted in reduced synthesis of proteoglycan and collagen, increased release of MMPs, accumulation of type II collagen fragments and higher branching of collagen fibrils. In contrast, reducing O_2 _during Phase II resulted in increased proteoglycan and type II collagen synthesis and reduced expression and release of MMP-13 mRNA and protein. Supplementation of cadmium chloride during differentiation culture at 5% O_2 _drastically reduced the up-regulation of type II collagen and the down-regulation of MMP-1 mRNA.

**Conclusions:**

The application of more physiologic oxygen percentage during specific phases of differentiation culture enhanced the biosynthetic activity and reduced the activity of catabolic enzymes implicated in cartilage breakdown. Modulation of the oxygen percentage during HAC culture may be used to study pathophysiological events occurring in osteoarthritis and to enhance properties of in vitro engineered cartilaginous tissues.

## Introduction

Homeostasis of normal cartilage in adults represents a delicate balance between the synthesis and the degradation of extracellular matrix components to maintain the functional integrity of the joint. In elderly individuals, together with changes in proliferation activity, energy metabolism and response to growth factors [1], chondrocytes become less resistant to extrinsic stress. This in turn causes a disturbance of tissue homeostasis and thus the risk of degenerative pathologies of osteoarthritic nature [2]. In particular the oxidative stress is proposed to play a key role in cartilage degeneration.

Oxygen is a critical parameter proposed to modulate chondrocyte metabolic activity [3]. Indeed, articular cartilage is generally exposed to a finely regulated gradient of relatively low oxygen percentages (from about 10% at the surface to about 1% in the deepest layers) [4], which is essential for maintenance of specialized tissue function [5]. During the onset of cartilage degeneration, possibly due to surface fibrillation and/or microfractures of the subchondral bone, such gradients have been proposed to break down [6], thus contributing to the progression of the disease.

The influence of various oxygen percentages on chondrocyte function has been investigated in a broad variety of models, differing with respect to (i) the cell source used (species: bovine, chicken, rodents, human, and anatomical locations of cell harvesting: knee, hip, interphalangeal joint, nose), (ii) the characteristic of the donor (age, stage of cartilage degeneration), (iii) the oxygen percentage applied (from less then 1% to more than 60%), (iv) the hydrodynamic culture conditions (static culture or mixing within bioreactors), and (v) the stage of cell differentiation (cells in native tissue, de-differentiated cells, re-differentiating expanded cells in pellets, alginate gels, or different types of porous scaffolds). It is thus not surprising that the data reported in literature on the influence of oxygen percentage on chondrocyte behavior are rather controversial [3]. For instance, as compared to culture under normoxic conditions (18 to 21% oxygen), culture at more physiological, *low *oxygen percentages (1 to 8%) has been reported to increase [7-10], decrease [11,12] or have no effect on the chondrocyte proliferation rate [6,13-15]. Moreover, the expression of cartilage specific genes and/or the extent of matrix protein synthesis/deposition was reported to be up-regulated [6-9,12,15-22], down-regulated [10,23-26] or not modulated at all [6,9] by culture under more physiological oxygen percentages.

Importantly, in addition to the still controversial findings, in the above mentioned studies the effect of oxygen percentage on chondrocytes has mainly been investigated with regard to the cell biosynthetic activity, without considering and exploring chondrocyte catabolic processes. We thus aimed our study at investigating the effect of a *low *(more physiological) oxygen percentage both on the cartilage tissue forming capacity of human articular chondrocytes (HAC), and on their pro-catabolic, matrix degradative activity. In particular, we hypothesized that culture at a more physiological oxygen percentage has a dual role in the chondrocyte metabolism, by enhancing their biosynthetic activity and at the same time reducing the expression of matrix degradative enzymes. To test these hypotheses, HAC were exposed to normoxic conditions (19%) or to a *low *oxygen percentage (5%) during culture in two simple and widely used model systems (that is, monolayer expansion or differentiation in micromass pellets), as well as at different phases of tissue development (that is., during de-novo tissue formation or in pre-formed tissues). We further investigated whether the applied oxygen percentage influences the structural organization of the collagen fibrils produced by HAC and whether those features have a pathophysiological counterpart in healthy and osteoarthritic cartilage tissue. Finally, in order to address whether the metabolic effects of HAC culture at *low *oxygen percentage involve signaling through the hypoxia-inducible factor-1α (HIF-1α) pathway, some cultures were supplemented with the specific inhibitor cadmium chloride.

## Materials and methods

### Cartilage samples collection

Macroscopically normal human articular cartilage samples (Mankin Score: 2 to 3) were obtained *post mortem *(within 24 hours after death) from the knee joints of a total of six donors with no clinical history of joint disorders (mean age: 56 years, range: 43 to 65 years), after informed consent by relatives and in accordance with the local ethics committee (University Hospital Basel, Switzerland). Cells from different donors were used for independent experimental runs. Osteoarthritic cartilage tissues (Mankin Score: 6 to 7) harvested from three patients undergoing total or partial knee replacement (female:male = 2:1, mean age: 67 years, range 65 to 71 years) were used as controls for degenerated structural organization of collagen fibrils.

### Chondrocyte isolation and expansion

Cartilage tissues were minced in small pieces and digested with 0.15% type II collagenase (10 ml solution/g tissue) for 22 hours. The isolated human articular chondrocytes (HAC) were expanded for two passages with Dulbecco's Eagle's Medium (DMEM) containing 4.5 mg/ml D-glucose, 0.1 mM nonessential amino acids, 1 mM sodium pyruvate, 100 mM HEPES buffer, 100 U/ml penicillin, 100 μg/ml streptomycin and 0.29 mg/ml L-glutamate supplemented with 10% of foetal bovine serum (*complete medium*) and 1 ng/ml of Transforming Growth Factor β1 (TGFβ-1), 5 ng/ml of Fibroblast Growth Factor 2, and 10 ng/mL of Platelet-Derived Growth Factor-BB (all from R&D Systems, Minneapolis, MN, USA) (*expansion medium*) [27] in a humidified incubator (37°C/5% CO_2_) at either normoxic condition (19% O_2_) or *low*, more physiological oxygen tension (5% O_2_). *Expansion medium *was equilibrated under 5% and 19% O_2 _for at least six hours before each media change. Expanded cells were subsequently cultivated in pellets as described below.

### 3D pellet cultures

The chondrogenic capacity of expanded HAC was investigated in pellet cultures under the two oxygen conditions (19% O_2 _and 5% O_2_) used for the expansion. Chondrocytes were re-suspended in *complete medium *supplemented with 10 μg/ml insulin (ACTRAPID HM), 0.1 mM ascorbic acid 2-phosphate (SIGMA, San Gallen, Switzerland), 10 ng/mL Transforming Growth Factor-β3 (Novartis, Basel, Switzerland) (*chondrogenic medium*) [27]. *Chondrogenic medium *was equilibrated under 5% and 19% O_2 _for at least six hours before each media change.

**Figure 1:**
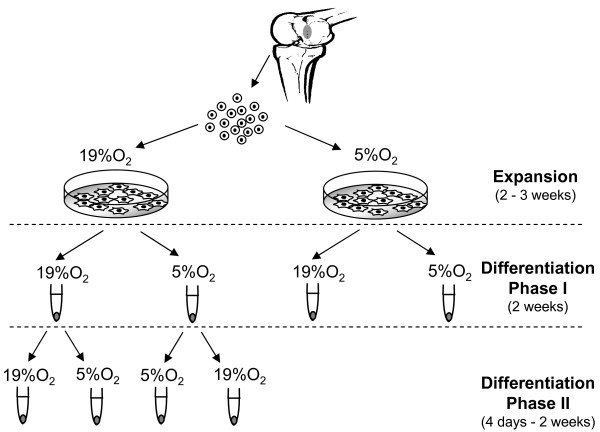
**Experimental design**. Human articular cartilage were cultured in monolayer (Expansion) under 5% and 19% oxygen percentages. Cells were then cultured for two weeks again under the two oxygen percentages (Differentiation Phase I). Pellets generated at 5% and 19% oxygen were further cultured at the same conditions or at interchanged oxygen percentages (Differentiation Phase II).

Pellets generated by cells from two donors after two weeks of culture under the two oxygen percentages (19% O_2 _or 5% O_2_) (Phase I) were further cultured for up to two weeks (Phase II) in *chondrogenic medium *at the same or at interchanged oxygen percentages (that is, from 5% to 19% O_2 _or from 19% to 5% O_2_) (Figure 1). For the HIF-1α inhibition experiments, pellets generated by cells from three donors after two weeks of culture at 19% O_2 _were subsequently exposed to 5% O_2 _and cultured for six hours or three days in *chondrogenic medium *supplemented with 5 μM cadmium chloride (CdCl_2_, SIGMA) [28].

Resulting tissues were analyzed histologically, immunohistochemically, biochemically and via scanning electronic microscopy to determine the quality of generated tissue, anabolic and catabolic cell functions and collagen fibril organization.

### Pellet characterization

#### Biochemical analyses

For the determination of the glycosaminoglycan (GAG) and DNA contents, pellets were digested with protease K (0.5 ml of 1 mg/ml protease K in 50 mM Tris with 1 mM EDTA, 1 mM iodoacetamide, and 10 μg/ml pepstatin-A for 15 hours at 56°C) as previously described [29]. GAG contents of pellets were measured spectrophotometrically using the dimethylmethylene blue (DMMB) assay [30]. The DNA amount was measured spectrofluorometrically using the CyQUANT^® ^Kit (Molecular Probes, Eugene, OR, USA) following the kit's instruction. GAG contents were reported as μg GAG/μg DNA.

#### Measurement of [^35^S]SO_4 _and [^3^H]proline incorporation

The proteoglycan and collagen synthesis of pellets were measured by assessing the incorporation of (^35^S)SO_4 _and (^3^H)proline for a period of 24 h as described previously [31]. Briefly, pellets were incubated in the presence of both (^35^S)SO_4 _(1 μCi/culture) to label proteoglycans and (^3^H)proline (1.5 μCi/culture) to label collagen. For the assessment of the released ECM fraction, radiolabeled proteoglycan and collagen were precipitated overnight at 4°C using respectively 100% ethanol and 70% ammonium sulphate and subsequently, resuspended in 4 M guanidine hydrochloride or 10% sodium dodecyl sulphate in Tris buffer (0.1 M, pH 7.0) respectively for proteoglycan and collagen. For the assessment of the incorporated ECM fraction, tissue pellets were digested with protease K as previously described. The incorporation of (^35^S)SO_4 _and (^3^H)proline in culture pellet and in conditioned medium was measured in a Packard β-liquid scintillation counter with scintillation fluid (Ultima Gold, Perkin Elmer, Schwerzenbach, Switzerland). The amount of synthesised molecules was normalized to the DNA content of the tissue.

#### Histological and immunohistochemical analyses

Pellets were fixed in 4% formalin, embedded in paraffin and cross-sectioned (5 μm thick sections). The sections were stained with Safranin O for sulfated GAG and processed for immunohistochemistry to visualize type II collagen (II-II6B3, Hybridoma Bank, University of Iowa, Iowa City, IA, USA), as described in Grogan et al. [32] and type II collagen fragments according to Roy-Beaudry et al. [33].

#### Electronic microscopy (SEM)

Images obtained from both scanning electron microscopy (SEM) and transmission electron microscopy (TEM) were used for the structural analysis of collagen fibrils. Pellet samples were glued onto a Teflon disc with a five-minute curing epoxy glue (Devcon Epoxy, ITW Brands, Wood Dale, IL, USA). After which, the mounted specimens were placed in a vibratory microtome (VT 1000 E, Leica, Heidelberg, Germany) to trim off the outermost, approximately 150 μm thick cartilage layer parallel to the support surface to minimize inhomogenities across the surface among samples. The surface layer of the adult healthy and OA cartilage was examined without any modification. The samples were then prepared for SEM and TEM analysis as previously described [34]. For TEM analysis, the samples were further homogenised into small pieces in order to isolate single collagen fibrils.

#### Image analysis

Quantitative data on the collagen fibril organization were obtained using the Image Processing Library & Toolbox (IPLT) image analysis software package (Basel, Switzerland) [35]. A Canny edge detection algorithm [36], followed by a skeletonization algorithm [37] was applied to identify the collagen fibrils. The skeletonized data were subjected to an algorithm identifying the end points and intersections of the skeleton. Using this information, the individual line segments were identified and analyzed. Finally, the following parameters were determined from each pellet condition: (i) the bending ratio, calculated as the mean-squared end-to-end distance divided by the mean-squared contour length and (ii) the persistence length, calculated using a previously described model [38]. Both these parameters were required to correlate the linearity of the fibrils and length before branching of each individual fibril to its mechanical properties, respectively [39].

#### Total RNA extraction and cDNA synthesis

Total RNA of pellets was extracted using Trizol (Life Technologies, Basel, Switzerland) and the standard single-step acid-phenol guanidinium method. RNA was treated with DNAseI using the DNA-free™ Kit (Ambion, Austin, Texas) and quantified spectrometrically. cDNA was generated from 3 μg of RNA by using 500 μg/ml random hexamers (Promega AG Dübendorf, Switzerland) and 1 μl of 50 U/ml Stratascript™ reverse transcriptase (Stratagene, Amsterdam, NL), in the presence of dNTPs. Real-time RT-PCR reactions were performed and monitored using the ABI Prism 7700 Sequence Detection System (Perkin-Elmer/Applied Biosystems, Rotkreuz, Switzerland). Cycle temperatures and times as well as primers and probes used for the reference gene (*18-S *rRNA) and the genes of interest (*collagen type II *and *aggrecan*) were as previously described [40]. Assays on-Demand (Applied Biosystem) were used to measure the expression of *MMP-1 *(Hs00233958_m1), *MMP-2 *(Hs00234422_m1), *MMP-9 *(Hs00234579_m1) and *MMP-13 *(Hs00233992_m1). For each cDNA sample, the threshold cycle (Ct) value of each target sequence was subtracted to the Ct value of *18-S *rRNA, to derive ΔCt. The level of gene expression was calculated as 2^ΔCt^. Each sample was assessed at least in duplicate for each gene of interest.

#### Quantification of released matrix metalloproteinases

Matrix metalloproteinases (MMP) were quantified in media collected from cultured pellets by using the MultiAnalyte Profiling MMP base Kit (Fluorokine^® ^MAP: LMP000) complemented with the specific MMPs (MMP-1: LMP901; MMP-3: LMP513; MMP-9: LMP911; MMP-13: LMP511, R&D Systems, Minneapolis, MN, USA). The assay was performed on a Luminex 100™ analyzer (Austin, Texas, USA) following the manufacturer's instructions. The amount of released MMPs was normalized to the DNA content of the tissue.

### Statistical analysis

For each analysis, triplicate pellets for each condition and donor were assessed. Statistical evaluation was performed using SPSS software version 7.5 software (SPSS, Sigma Stat, Erkrath, Germany). Values are presented as mean ± standard deviation (SD). Differences between groups were assessed by Mann Whitney tests. Differences in the persistence length and bending ratio of collagen fibrils from different conditions were assessed by one-way analysis of variance (ANOVA) with Bonferroni post hoc test. Values of *P *< 0.05 were considered statistically significant.

## Results

### Chondrogenic differentiation of HAC cultured under different oxygen percentages

**Figure 2:**
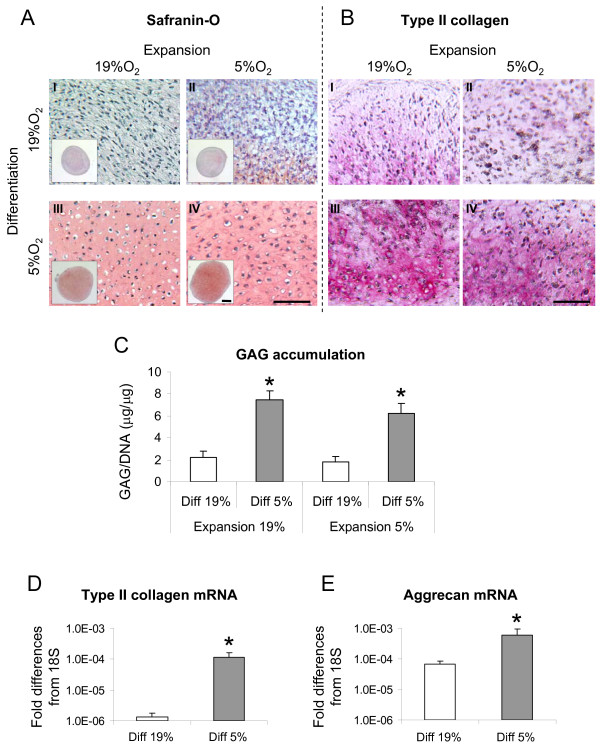
**Anabolic response of HAC to different oxygen percentages during the expansion and differentiation Phase I**. **(A - B) **Safranin O and type II collagen immunohistochemical stainings of representative tissues generated by human articular chondrocytes (HAC) expanded at 19% (**I **and **III**) or 5% (**II **and **IV**) oxygen and further cultured in pellets at 19% (**I **and **II**) or 5% (**III **and **IV**) oxygen. Bar = 100 μm. (**C**) Quantification of glycosaminoglycans (GAG) accumulated normalized to the amount of DNA. (**D **- **E**) Real time reverse transcription-polymerase chain reaction analysis of the expression of *type II collagen *and *aggrecan *mRNA by HAC cultured in pellets at 19% and 5% O_2_. Levels are expressed as fold of difference from ribosomal 18S. For the gene expression analysis only expansion at 19% O_2 _was considered. Values are mean ± SD of measurements obtained from three independent experiments. * = significantly different from the 19% O_2_.

HAC were initially cultured in monolayer with *expansion medium *at 5% or 19% O_2 _and subsequently re-differentiated in three-dimensional pellets at the two different oxygen percentages (Phase I) (See Figure 1 for the experimental design). HAC proliferated at comparable rates (less than 5% variation in the number of doublings/day; data not shown) at the two oxygen conditions. Cells expanded at either oxygen percentage and subsequently differentiated at 19% O_2 _produced tissues faintly stained for GAG and type II collagen (Figures 2A, I and 2II and 2B, I and 2II). Instead, reducing oxygen percentage during differentiation enhanced the amount of cartilaginous matrix accumulation, as evidenced by a qualitative increased size of the generated tissues (Figure 2A, low magnification), an increased intensity of Safranin O and type II collagen stain (Figure 2A, B) and a statistically significant higher amount of GAG (3.4- and 3.1-fold for HAC expanded at 19% or 5% O_2 _respectively) (Figure 2C). Due to the fact that expansion at 5% O_2 _did not influence the extent of HAC differentiation, further assessments were only performed with cells expanded at 19% O_2_. In agreement with the histological and biochemical results, the RT-PCR analysis confirmed statistically significant higher expression of the cartilage specific genes *type II collagen *(86.6-fold) and *aggrecan *(8.5-fold) at 5% O_2 _than at 19% O_2 _after the Phase I differentiation culture (Figure 2D, E).

### Expression of catabolic mediators

**Figure 3:**
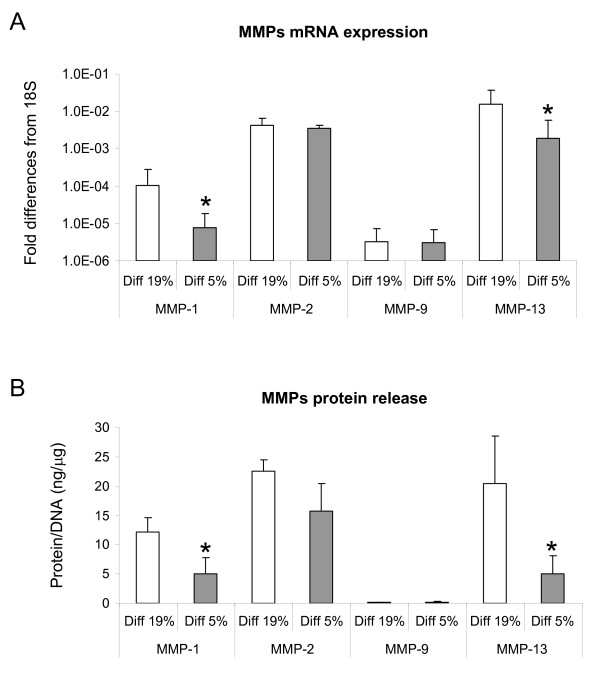
**Quantification of MMPs produced by HAC cultured at different oxygen percentages during the Phase I**. (**A**) Real time reverse transcription-polymerase chain reaction analysis of the expression of MMP-1, -2, -9, -13 mRNA by human articular chondrocytes (HAC) cultured in pellets at 19% and 5% O_2_. Levels are expressed as fold of difference from ribosomal 18S. (**B**) Quantification of MMP-1, -2, -9, -13 released in the culture medium. Levels are normalized to the amount of DNA measured in relative pellets. Values are mean ± SD of measurements obtained from three independent experiments. * = significantly different from the 19% O_2_.

We then investigated the possible role of oxygen percentage in modulating the expression of catabolic mediators. Analysis of specific matrix metalloproteinases (that is, *MMP-1*, *MMP-2*, *MMP-9 *and *MMP-13*) by RT-PCR indicated that low oxygen percentage applied during the Phase I differentiation culture selectively down-regulated *MMP-1 *and *MMP-13 *mRNA expression (7.7- and 3.5-fold, respectively). *MMP-2 *mRNA was highly expressed and not modulated by the oxygen percentage. The expression of *MMP-9 *mRNA remained unaffected and was at the limit of detection at both oxygen percentages (Figure 3A).

The protein levels of MMP-1, -2, -9, -13 were assessed in the supernatant of pellet cultures at the end of Phase I. Consistent with the mRNA results, the amounts of MMP-1 and -13 released were reduced in the pellets cultured at 5% O_2 _as compared to those cultured at 19% O_2 _(8.2- and 11.3-fold respectively). The protein expression levels of MMP-2 and -9 remained similar at the different oxygen percentages (Figure 3B).

### Effect of oxygen percentage on HAC anabolic and catabolic activity in pre-formed cartilaginous tissues

We next investigated the influence of oxygen in anabolic (synthesis and accumulation of cartilaginous matrix proteins) and catabolic (MMPs expression, activity and degradation products) processes of pre-formed tissues. Pellets generated after two weeks of culture at 19% O_2 _or 5% O_2 _(Phase I) were subsequently cultured up to an additional two weeks (Phase II) at the same or at interchanged oxygen percentages (Figure 1).

#### Accumulation and synthesis of cartilaginous matrix proteins

**Figure 4:**
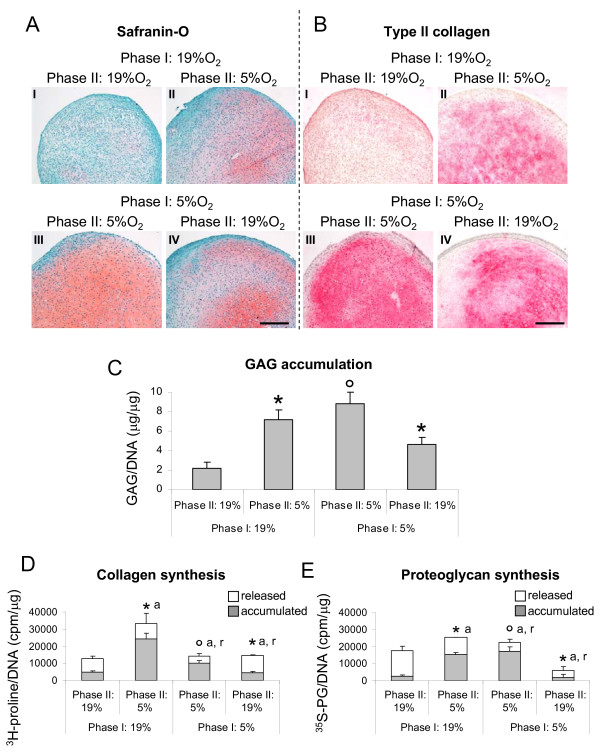
**Anabolic response of HAC to different oxygen percentages during differentiation Phase I and II**. (**A **- **B**) Safranin O and type II collagen stainings of representative tissues generated by human articular chondrocytes (HAC) cultured in pellets for two weeks (Phase I) at 19% (**I **and **II**) or 5% (**III **and **IV**) oxygen and further cultured for two additionally weeks (Phase II) at 19% (**I **and **III**) or 5% (**II **and **IV**) oxygen. Bar = 100 μm. (**C**) Quantification of glycosaminoglycans (GAG) accumulated in pellets cultured as described in (A - B) normalized to the amount of DNA. (**D **- **E**) Amounts of newly synthesized collagen (D) and proteoglycan (E) measured in pellets cultured for 18 days (two weeks of Phase I and four days of Phase II). The upper and lower parts of the columns represent the released and accumulated fractions respectively. Values are mean ± SD of measurements obtained from two independent experiments. * = significantly different from the group cultured with the same oxygen percentage in Phase I but with different oxygen tension in Phase II; ° = significantly different from the group cultured entirely at 19% O_2_; a = accumulated, r = released.

In agreement with the above described results, pellets cultured for four weeks (two weeks of Phase I and two weeks of Phase II) at 5% O_2 _were more strongly stained for Safranin O and type II collagen, and accumulated larger amounts of GAG (4.0-fold) as compared to those cultured for the same time at 19% O_2 _(Figure 4A, B, C). Reducing oxygen percentage during Phase II for pellets cultured at 19% during Phase I resulted in an improved quality of the cartilaginous tissues, as assessed by an increased accumulation of cartilaginous matrix positive for GAG and type II collagen (Figure 4A, B) and by a higher GAG content (3.3-fold) (Figure 4C). Conversely, increasing oxygen percentage during Phase II for pellets cultured at 5% during Phase I resulted in a reduced accumulation of cartilaginous matrix (Figure 4A, B) and GAG content (1.9-fold) (Figure 4C).

Results from the radiolabelling experiments indicated that similar amounts of total collagen and proteoglycan (that is, released + accumulated) were synthesized by pellets cultured for 18 days (two weeks of Phase I and four days of Phase II) at the two oxygen percentages. However, as compared to 19% oxygen (Phase I and Phase II), the released fractions of these newly synthesized macromolecules by pellets cultured at 5% O_2 _(Phase I and Phase II) were markedly and statistically significantly lower (2.0- and 2.9-fold respectively for collagen and proteoglycan), while the accumulated fractions were higher (2.1- and 6.6-fold respectively for collagen and proteoglycan). Consistent with the biochemical results, the culture at 5% O_2 _during Phase II of tissues pre-formed at 19% O_2 _during Phase I resulted in an augmented synthesis of collagen and proteoglycan (respectively by 2.7- and 1.4-fold). In particular, the increased synthesis of the newly synthesized macromolecules was mainly reflected by an augmented accumulation (up to 5.9-fold). Instead, the culture at 19% O_2 _during Phase II of tissues pre-formed at 5% O_2 _during Phase I differently modulated the synthesis of the two extracellular matrix molecules: while a decreased accumulation (2.3-fold) and an increased released (2.6-fold) was measured for collagen, only a reduction of the accumulated fraction was demonstrated for proteoglycan (8.6-fold) (Figure 4D, E).

#### MMPs production and activity

**Figure 5:**
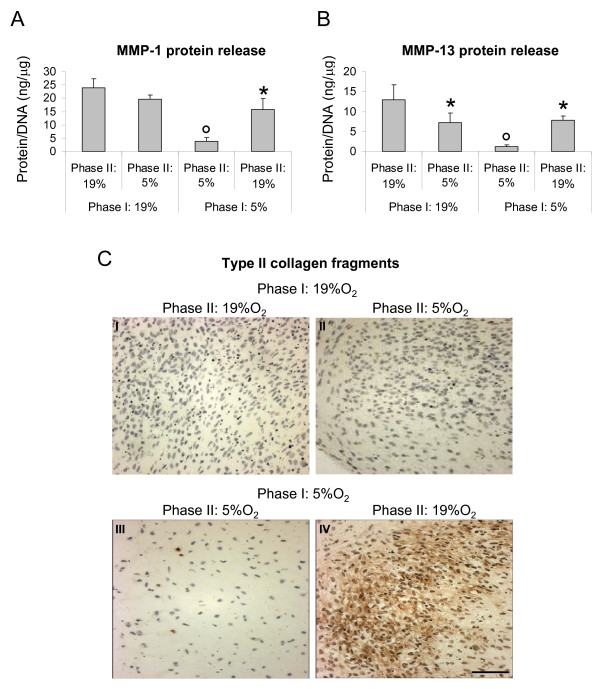
**Catabolic response of HAC to different oxygen percentages during differentiation Phase I and II**. (**A **- **B**) Quantification of MMP-1 (**A**) and MMP-13 (**B**) released in the medium by human articular chondrocytes (HAC) cultured in pellets for four weeks (two weeks of Phase I and two weeks of Phase II). Levels are normalized to the amount of DNA measured in relative pellets. Values are mean ± SD of measurements obtained from two independent experiments. * = significantly different from the group cultured with the same oxygen percentage in Phase I but with different oxygen tension in Phase II; ° = significantly different from the group cultured entirely at 19% O_2 _(Phase I and Phase II). (**C**) Immunohistochemical detection of type II collagen fragments of pellets cultured under conditions described in (A - B). Bar = 100 μm.

Pellets cultured for four weeks (two weeks of Phase I and two weeks of Phase II) at 5% O_2 _released lower amounts of MMP-1 and -13 (6.1- and 10.1-fold respectively) as compared to those cultured for the same time at 19% O_2_. Culture at 5% O_2 _during Phase II of tissues pre-formed at 19% O_2 _during Phase I resulted in reduced production of both MMPs, though only MMP-13 by statistically significant levels (by 1.8-fold). Instead, culture at 19% O_2 _during Phase II of pellets pre-formed at 5% O_2 _during Phase I resulted in increased release of both MMP-1 and MMP-13 (4.0- and 6.2-fold respectively) (Figure 5A, B).

In order to assess whether the observed increased production of MMPs corresponded to an increased proteinase activity, pellets cultured for a total of four weeks at the different oxygen percentages were assessed immunohistochemically to detect the presence of type II collagen C-telopeptides, derived by MMP-1 and -13 collagenolytic activity [33]. Analyses indicated that only the pellets formed at 5% O_2 _during Phase I and subsequently cultured at 19% O_2 _during Phase II were intensely stained for the type II collagen fragments (Figure 5C).

#### Collagen fibril organization

**Figure 6:**
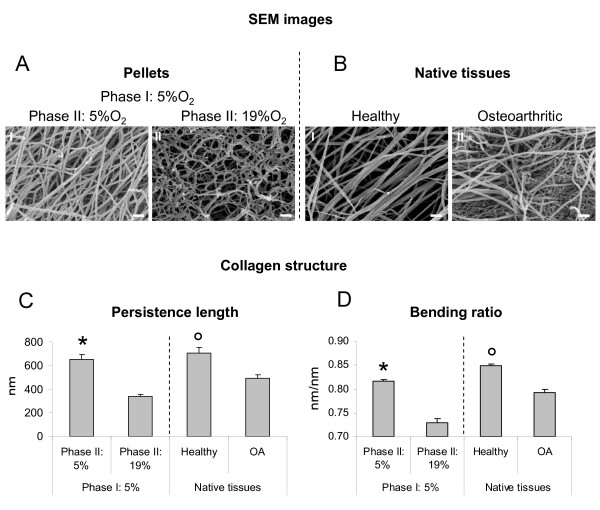
**SEM images and structural analysis of extracellular collagen-fibrils from engineered, healthy and osteoarthritic cartilage samples**. Representative scanning electron microscopy (SEM) images of (**A**) tissues generated by culturing human articular chondrocytes in pellets for four weeks (two weeks of Phase I and two weeks of Phase II) or (**B**) native human tissue biopsies from healthy or osteoarthritic (OA) cartilage. (**C**) Persistence length and (**D**) bending ratio assessment of the extracellular fibril network of engineered and native tissues. * = significant different from 19% O_2_; ° = significant different from OA tissues.

To determine whether increasing oxygen percentage during cultivation Phase II of tissues pre-formed at 5% O_2 _would change the structure and arrangement of the collagen fibril network, pellets were qualitatively and quantitatively assessed via EM. Images indicated that the collagen fibrils of pellets cultured at 5% O_2 _during Phase I and then for two weeks at 19% O_2 _during Phase II were less linear than those of pellets cultured for four weeks at 5% O_2_. Interestingly, a similar trend was also observed in the OA cartilage as compared to healthy cartilage samples (Figure 6A, B). In pellets, the collagen network was comprised of single fibrils with diameters ranging from 20 to 30 nm. In healthy adult cartilage, the network contained bundled and twisted collagen fibrils three- to four-fold larger in diameter. Quantitative image analysis indicated that increasing the oxygen percentage during Phase II resulted in a significant reduction of persistence length as well as bending ratio (47.9% and 10.5% respectively). Interestingly, both parameters were higher in healthy as compared to OA tissues (30.0% and 6.6% respectively for persistence length and bending ratio). Considerable decrease in persistence length and bending ratio would indicate softening and gradual deterioration of cartilage physiological function [39].

#### Response to low oxygen under CdCl_2_-treatment

**Figure 7:**
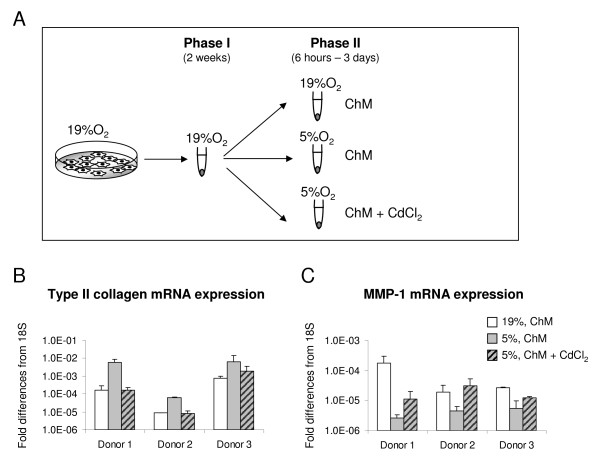
**Effects inhibition of HIF-1α on anabolic and catabolic gene regulation at *low *oxygen percentage**. (**A**) Experimental design: human articular chondrocytes from three donors were cultured as pellets in *chondrogenic medium *(ChM) at 19% O_2 _(Phase I) and subsequently maintained at the same oxygen percentage or exposed to 5% O_2 _in the absence or presence of 5 μM CdCl_2 _for six hours or three days (Phase II). Real time reverse transcription-polymerase chain reaction analysis of *type II collagen *mRNA expression after six hours (**B**) and of *MMP-1 *mRNA expression after three days (**C**). Levels are expressed as fold of difference from ribosomal 18S. Values for each donor are mean ± SD of measurements obtained from three independent pellets.

To determine whether the observed pro-anabolic and anti-catabolic effects of low oxygen percentage are mediated by HIF-1α, HAC from three donors were pre-cultured in pellets during Phase I at 19% O_2_. During the subsequent culture Phase II, the pre-cultured pellets were maintained at 19% O_2 _or exposed to 5% O_2_, with or without treatment with CdCl_2 _for six hours or three days (Figure 7A). Following culture at *low *oxygen percentage, *type II collagen *mRNA was up-regulated to a higher extent after six hours (up to 33.0-fold; Figure 7B) than after three days (data not shown), while *MMP-1 *mRNA was down-regulated to a higher extent after three days (up to 65.5-fold; Figure 7C) than after six hours (data not shown). Supplementation of CdCl_2 _during this culture phase almost abrogated the aforementioned *low *O_2_-mediated effects, so that the expression of *type II collagen *and *MMP-1 *mRNA reached levels comparable to those of cells cultured at 19% O_2 _for the corresponding times (Figure 7B, C).

## Discussion

In this study we found that culture at *low*, more physiological (5%) oxygen percentage has a dual role in HAC metabolism, namely to enhance the proteoglycan and collagen synthesis and at the same time to reduce the activity of two key catabolic enzymes involved in cartilage breakdown (that is, MMP-1 and MMP-13). As a consequence, HAC exposure to 19% oxygen reduced the de novo formation of cartilage tissue and induced degradation of pre-deposited collagen fibrils, leading to structural features similar to those found in osteoarthritic tissue. Interestingly, HAC appeared to be highly sensitive to the oxygen percentage applied during differentiation culture in pellets, but not during expansion in monolayers. The anti-anabolic and pro-catabolic effects mediated by *low *oxygen percentage were HIF1α-dependent, as assessed by specific inhibition of this factor by CdCl_2 _treatment.

The application of 5% oxygen percentage during the HAC monolayer expansion did not influence the proliferation rate and chondrogenic capacity of HAC. This is in contrast with results reported by Egli et al. [7], indicating that *bovine* articular chondrocytes expanded under hypoxic conditions generated tissues with higher amounts of cartilaginous matrix as compared to those expanded under normoxic conditions. The discrepancy between our results and those generated by Egli et al. [7] can be related to the different type of cells used (human vs bovine), the stage of cell de-differentiation (second passaged vs first passaged cells) and/or the specific oxygen percentage tested (5% vs 1.5%). Indeed, HAC culture at lower than 5% oxygen during expansion may lead to a benefit in their redifferentiation capacity, and remains to be investigated.

The influence of oxygen percentage during the de-novo tissue formation was evaluated by culturing HAC in micromass pellets, a model commonly used to investigate in vitro cartilage development. Our results indicate that the application of 5% as compared to 19% oxygen percentage critically enhanced the chondrogenic capacity of HAC, as assessed by a greater accumulation of GAG and type II collagen. Similar responses to reduced oxygen percentage have been reported [9] using human nasal chondrocytes statically cultured in pellets for three days and subsequently transferred to a dynamic bioreactor system. We also investigated whether culture of chondrocytes at *low *oxygen percentage modulated the production of specific metalloproteinases involved in the degradation of extracellular matrix proteins. We observed that the expression of MMP-1 and MMP-13, both at mRNA and protein levels, was reduced in cells cultured at 5% as compared to 19% oxygen. Interestingly, MMP-1 (or collagenase-1) and/or MMP-13 (or collagenase-3) are among the enzymes expressed by human chondrocytes in degenerative pathologies of cartilage, namely osteoarthritis and rheumatoid arthritis [41] and are thus thought to play a critical role in cartilage destruction. In particular, it has been shown that both MMPs are involved in the initial phase of type II collagen breakdown [42,43], and MMP-13 is the collagenase with highest affinity for type II collagen [44]. However, the expression of other MMPs or degradative enzymes (for example, aggrecanases) not included in our study might also be regulated by culture at *low *oxygen tension.

Our results prompted us to hypothesize that different oxygen percentages could regulate not only cartilage generation, but also its further maturation and stability. We thus exposed tissues formed at the different oxygen percentages for two weeks (Phase I) to interchanged oxygen percentages in a subsequent culture phase (Phase II). Results obtained from the radiolabelling experiments indicated that the exposure of tissues to 5% oxygen during Phase II induced higher synthesis and accumulation of collagen and proteoglycan. It remains to be assessed whether low oxygen percentages also enhance expression of molecules involved in stabilization of the newly synthesized extracellular matrix components (for example, decorin, fibromodulin, link protein, type IX collagen) [45]. Importantly, the presence of type II collagen cleavage products, indicative of MMP activity, was immunohistochemically detected [33] only in the pellets pre-formed at 5% oxygen (Phase I) and subsequently cultured for additional two weeks at 19% oxygen (Phase II). These results, together with the observed enhanced expression of MMP-1 and -13 at 19% oxygen, strongly indicate a direct involvement of oxygen in regulating the MMP-mediated breakdown of cartilaginous tissues. The result that pellets entirely cultured at 19% O_2 _negatively stained for type II collagen fragments could be explained by the insufficient accumulation of the MMP substrate (that is, type II collagen) during the initial cultivation Phase I.

The presence of type II collagen fragments correlated well with the branched/tangled collagen fibril organization and decreased values of bending ratio and persistence length in pellets exposed to 19% oxygen. This could possibly result from an increased enzymatic cleavage of the extracellular matrix molecules by specific MMPs. Conclusively, increased activity of catabolic enzymes is affecting the collagen fibril network that exhibits lower values of bending ratio and persistence length. Based on this correlation, both parameters could potentially represent valuable markers for determining the degree of collagen deterioration. Exposure of cartilage tissues formed at physiological oxygen percentages to higher oxygen levels resembled degradation events occurring during the progression of OA, where, following initial pathologic events, the normal oxygen gradients break down [6]. Therefore, our tissue engineering model would be instrumental to investigation of the evolution of cartilage damage following alteration of the oxygen levels and to assess the effect of possible therapeutic targets.

The observed pro-anabolic and anti-catabolic effects of *low *oxygen culture were mediated by the hypoxia inducible signaling pathway, since reduction of the oxygen percentage did not regulate type II collagen and MMP-1 mRNA expression in the presence of the HIF-1α inhibitor cadmium chloride (CdCl_2_) [28]. While the importance of HIF-1α in modulating the expression/synthesis of cartilage-specific genes was recently addressed [28-46], the involvement of this factor in the oxygen-dependent modulation of catabolic genes, recently reported for porcine pulmonary artery endothelial and smooth muscle cells [47], has not been previously postulated for HAC.

## Conclusions

The present study demonstrates that *low *oxygen percentage applied during the differentiation phases of human articular chondrocyte culture enhances cell biosynthetic activity as well as reduces the activity of catabolic enzymes known to play key roles in the breakdown of cartilage matrix during degenerative pathologies. These findings indicate that regulation of oxygen percentages during *in vitro *culture could be used to improve the generation of functional cartilage substitutes, and thus prompt the development of tools enabling accurate control of oxygen levels for tissues of clinically relevant size [48]. Moreover, modulation of oxygen tension in cultured HAC may be used as a tool to model and study *in vitro *pathophysiological events occurring in osteoarthritis. Finally, following such investigations, the identification of innovative strategies to maintain local *in vivo *oxygen percentages to defined levels could represent a powerful tool for preventing the progression of degenerative cartilage diseases.

## Abbreviations

ANOVA: analysis of variance; cDNA: complementary deoxyribonucleic acid; CO_2_: carbon dioxide; Ct: threshold cycle; DMEM: Dulbecco's modified Eagle's medium; DMMB: dimethylmethylene blue; dNTP: deoxyribonucleotide; ECM: extracellular matrix; EDTA: ethylenediaminetetraacetic acid; EM: electronic microscopy; GAG: glycosaminoglycans; HAC: human articular chondrocytes; HEPES: 4-(2-hydroxyethyl)-1-piperazineethanesulfonic acid; HIF-1α: hypoxia-inducible factor-1alpha; IPLT: Image Processing Library & Toolbox; MMP: metalloproteinase; mRNA: messenger ribonucleic acid; O_2_: oxygen; OA: osteoarthritis; PBS: phosphate buffered saline; RNA: ribonucleic acid; rRNA: ribosomal ribonucleic acid; RT-PCR: reverse-transcriptase polymerase chain reaction; SD: standard deviation; SEM: scanning electron microscopy; TEM: transmission electron microscopy; TGFβ1: transforming growth factor beta-1.

## Competing interests

The authors declare that they have no competing interests.

## Authors' contributions

SS participated in study conception and design, acquisition of data (biochemistry, histology, immunohistochemistry for type II collagen, RT-PCR analysis and cell culture), in the study design, in the interpretation of data and drafting the manuscript. ML participated in acquisition of the data (scanning electronic microscopy and image analysis) and in the interpretation of data. DW participated in study conception in the study design and revised the manuscript. ADS participated in analysis (image analysis). CC participated in study conception and provided the patient biopsies and their clinical data. RLPL participated in the development of the Luminex assays. FM participated in the acquisition of data (immunohistochemistry for type II collagen fragments) and revised the manuscript. AB and IM were responsible for study design, supervision of the experiments, interpretation of data and participated in writing the manuscript. All authors read and approved the final manuscript.
